# Subjective and objective components of resource value additively increase aggression in parasitoid contests

**DOI:** 10.1098/rsbl.2013.0391

**Published:** 2013-08-23

**Authors:** Bernard C. Stockermans, Ian C. W. Hardy

**Affiliations:** School of Biosciences, University of Nottingham, Sutton Bonington Campus, Loughborough, Leicestershire LE12 5RD, UK

**Keywords:** aggression, contests, resource value, contestant age, host size, parasitoid

## Abstract

Two major categories of factors are predicted to influence behaviour in dyadic contests; differences in the abilities of the contestants to acquire and retain resources (resource holding potential), and the value of the contested resource (resource value, RV; which comprises objective and subjective components). Recent studies indicate that subjective components affect contest behaviour in several animal taxa but few have simultaneously investigated objective RV components. We find that both an objective (host size) and a subjective (contestant age) component of RV affect contest intensity in the parasitoid wasp *Goniozus legneri.* These additively influence aggressiveness, with a larger effect from the subjective component than the objective component. The greater influence of subjective RV adds weight to the recent surge of recognition of this RV component's importance in contest behaviour.

## 1. Introduction

Animal contests over indivisible resources have been the subject of game theoretical scrutiny since the foundation of evolutionary behavioural ecology and their study has played a major part in the further development of the field [1,2]. Models predict that two major categories of factors influence behaviour in dyadic contests; those associated with the difference in the abilities of the contestants to acquire and retain resources (resource holding potential, RHP; [1,2]), and those associated with resource value (RV; [1,2]). RHP and RV may influence contests concurrently and both can be subdivided into constituent components. RHP may consist of the fundamental ability of contestants, such as physical strength (‘fighting ability’), modified by further influences on contest ability, for example, positional advantages and prior-ownership. RV may comprise many aspects that contribute to its overall value [2,3].

While contestants might be expected to compete more intensively for a higher value resource [2], the value that each contestant places on a given resource may be different [2,3]: there are thus objective and subjective components to RV. Objective aspects are properties intrinsic to the resource, which can be detected by the evaluator and will yield a certain fitness gain if obtained [4,5]. For instance, larger food items have objectively higher value than smaller items. Subjective aspects derive from a variety of different circumstances that may increase a given resource's value to an individual, causing it to take more risks, fight harder and expend more energy to acquire or defend it, than would competitors that have experienced different circumstances. For instance, food items of a given size have greater subjective value to hungry competitors than to satiated competitors [3]. Recent empirical studies indicate that the subjective value of resources affects contests in a several taxa [4], with subjective RVs being contingent on prior experience of resource-poor environments [5–7], investment in exploiting a resource [4,5,8] or experience of winning contests [9]. Although such studies cover a variety of subjective RV components, most do not simultaneously investigate objective RV components (exceptions include [4,10,11]).

We explore the influences of an objective and a subjective component of RV on the intensity of contest behaviour. We use *Goniozus legneri*, a parasitoid in which adult females compete agonistically for hosts: *Goniozus* contests are influenced by RHP (contestant size) and by several components of RV, including host size, host age, contestant age and the developmental stage of offspring [7]. Host size (quantity) and host age (quality) will typically form objective components of RV, whereas the age of competitors and their respective reproductive states constitute subjective influences on the value they are likely to place on host possession. We focus on the effects of host size (more offspring can be produced from larger hosts [7]) and contestant age, and their potential interaction, while minimizing or removing other asymmetries known to affect *Goniozus* contests [7,10–12]. The reason for assessing age effects is that older contestants should value more greatly an opportunity to reproduce, because their estimate of environment quality, in terms of the probability of finding a host, will be lower than that of a contestant that has obtained a host at a younger age [2,7,10]. We assess contest behaviour in terms of the level of aggression during dyadic encounters [12].

## 2. Material and methods

*Goniozus legneri* Gordh (Hymenoptera: Bethylidae) were of the same strain used in prior studies [12], and were reared on larvae of the moth *Corcyra cephalonica* Stainton (Lepidoptera: Pyralidae) [12]. Experiments and cultures were kept under constant illumination at 27°C and high relative humidity.

Objective value varied by providing each female with either a ‘small’ or a ‘large’ host the day prior to the contest (hosts were weighed to ± 0.01 mg; ‘small’ mean body mass ± s.d. = 29.25 ± 3.28 mg; ‘large’ = 53.11 ± 4.16 mg). Subjective value was manipulated by using females that were either ‘young’ (1–2 days post-eclosion from their pupal cocoons) or ‘old’ (5–7 days). *Goniozus legneri* adults typically live around 10 days under laboratory conditions.

Contest dyads were created in a two-way factorial design for contestant age and host size categories, with four possible combinations: two old wasps each with a large host (old–large), two old wasps each with a small host (old–small), two young wasps each with a large host (young–large) and two young wasps each with a small host (young–small). There were no categorical asymmetries in host size or contestant age within any replicate: within-replicate asymmetries in the actual ages of wasps and host sizes were minimized by pairing contestants that were within 24 h of being the same age and had hosts within 1.00 mg of being the same mass. Wasps were weighed to ± 0.01 mg on the day before the contest and contestants within each replicate chosen to be of closely similar mass ( ± 0.08 mg), so as to minimize size asymmetries that are known to affect contests [7,10–12]. A dyad never consisted of brood-mate females. Wasps had no prior contest experience. Using pairs of females that were both initially in possession of a host removed prior-ownership asymmetry, which influences owner–intruder contests [7,10–12].

Contests were observed in experimental arenas [12]. Prior to the contest, each female plus its host was isolated from the other by a barrier across the arena. Encounters and contest behaviour occurred once the barrier was opened. Behavioural interactions were recorded on videotape from above for 10 min following the first interaction and scored using Jwatcher v. 1.0. Interactions between contestants were categorized as: non-aggressive, chase, bite, sting and fight (mutual grappling), with the latter four considered to be aggressive [12]. The response variable was the proportion of interactions that were aggressive out of the total interactions in each replicate [12]. There were 79 replicates: 21 old–large; 18 old–small; 20 young–large; 20 young–small. There were also three ‘unsuccessful’ replicates where the wasps did not visibly interact at all (qualitatively different from ‘non-aggressive interaction’): these were excluded from the analyses. Data are available in the electronic supplementary material.

The influences of host size and contestant age categories on the proportion of behaviours that were aggressive were explored using logistic analysis (GenStat v. 14.1, VSN International Ltd.), with backwards elimination of explanatory variables and with overdispersion taken into account via empirical estimation of scaling parameters [13]. Because within-replicate size differences between contestant females are very difficult to eliminate completely, the relative mass difference between contestants was also included in the initial statistical model.

## 3. Results

**Figure 1.**
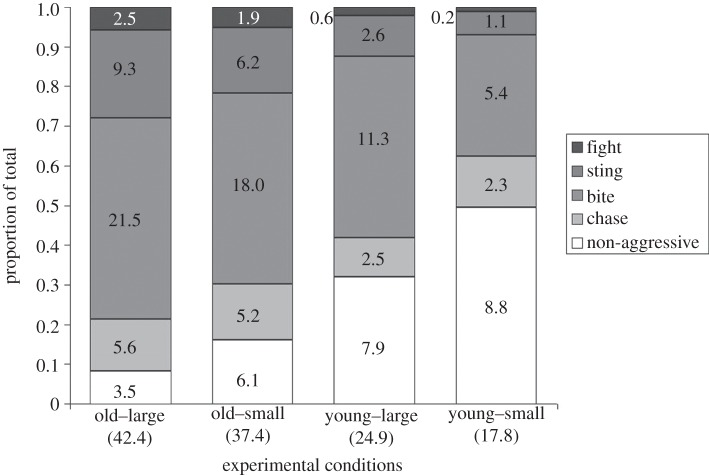
Effects of host size and wasp age on the proportions of each type of behaviour. Behaviours are stacked according to apparent escalation towards fighting [14]. Average per replicate occurrences of each behaviour are shown. Average per replicate total numbers of interactions are given in parentheses.

**Figure 2.**
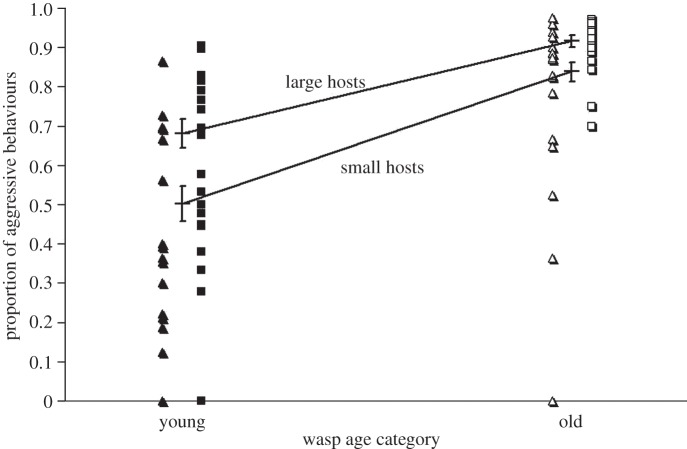
Effects of wasp age and host size on the proportion of aggressive behaviours. The means for each of the categorical combinations are shown by crosses linked by lines: the convergence of these lines is due to back-transformation of estimated proportions from the logit scale (on which they are parallel) and not due to statistical interactions (which were non-significant, table 1). Filled triangle, small host; filled square, large host; open symbols, old wasps; closed symbols, young wasps.

There were 1–88 behavioural interactions per dyad (mean ± s.d.: 30.49 ± 20.26). The most active contests occurred between old wasps competing for large hosts and the least active were between young wasps competing for small hosts (figure 1). Overall, 78.62 per cent (+ s.e. = 2.18%, − s.e. = 2.36%) of interactions were aggressive. The proportion of interactions that were aggressive was influenced by both host size and wasp age, but not by difference in wasp size or any statistical interactions between these main effects (table 1; figures 1 and 2). The most aggressive contests occurred between old wasps competing for large hosts and the least aggressive between young wasps competing for small hosts (figures 1 and 2). Aggressive behaviours can be ranked according to apparent escalation towards full fighting [14], and the more escalated behaviours were more common when the overall proportion of aggression was greater (figure 1), indicating that both the proportion and the intensity of aggression were affected by wasp age and host size. Given that both wasp age and host size were varied widely, we can conclude [15] that the effect of wasp age on aggression was greater (more deviance explained (table 1) and greater differences in aggression observed (figure 2)) than the effect of host size.

**Table 1.** Logistic analysis of influences on the proportion of aggressive behaviours.

source	d.f.	deviance	*F*	*p*	% deviance explained
wasp age	1	248.831	64.46	<0.001	42.94
host size	1	50.106	12.98	<0.001	8.65
wasp size difference	1	0.649	0.17	0.683	0.11
host size × wasp age interaction	1	0.083	0.02	0.884	0.01
wasp size difference × wasp age interaction	1	0.274	0.07	0.791	0.05
wasp size difference × host size interaction	1	5.436	1.41	0.239	0.94
wasp size difference × host size × wasp age interaction	1	8.350	2.16	0.146	1.44
residual	71	274.057			
total	78	579.504

## 4. Discussion

Contestant age and host size influence aggressiveness of parasitoid contest behaviour. Because larger hosts yield more offspring, host size is a correlate of host quality or objective RV [7,10], and it is unsurprising that females competed more aggressively for larger hosts. Older contestants also behaved more aggressively: we interpret this as being due to older females evaluating the probability of obtaining a host as lower than that of younger females and thus valuing their hosts more highly (subjective RV). RHP-based explanations would require RHP to increase with age, owing to contest experience or physiological development. As females were naive contestants, the former is excluded and, as organisms in the final phases of their life cycles are not typically at physiological prime, the latter appears quite unlikely [10]. We thus consider that RV influences the aggressiveness of parasitoid contest behaviour via both a subjective and an objective component. The effects of these components are additive not interactive, which should make straightforward the formulation of models predicting the influence of RV on aggression during contests and their outcomes, as, on current evidence, total RV can be formulated simply as the sum of its parts.

Subjective RV effects have been found across a range of taxa [3], including parasitoids [5,7,10,11,16]. The greater importance of the subjective component that we evaluated accords with recent findings that subjective RV is the predominant influence on contest outcomes in the parasitoids *Eupelmus vuilleti* and *Dinarmus basalis* [5,16]. In these species, subjective RV effects operate via egg maturation state, whereas egg load effects have only been found in *Goniozus* when other asymmetries were absent [7]. Further, subjective RV appears responsible for a ‘winner effect’ in *E. vuilleti* [9], contrasting with previous theory, and evidence from non-parasitoid taxa, that winner–loser effects are influenced by RHP changes [7,17]. In *G. legneri* as well, winner–loser effects appear owing to changes in RHP [7].

In summary, both objective and subjective components of RV affect contest behaviour simultaneously and additively. The greater influence of subjective RV adds weight to the recent surge of recognition [3,5,9,16] afforded to this component of the value individuals place on the resources they contest.
